# Fusion Revision Surgery With Reamer-Irrigator-Aspirator to Harvest Autograft After Spinal Pseudarthrosis

**DOI:** 10.7759/cureus.27503

**Published:** 2022-07-31

**Authors:** Ahmed H Elhessy, Hady H Eltayeby, Stephen C Kane, Ira M Garonzik, James E Conway, Janet D Conway

**Affiliations:** 1 International Center for Limb Lengthening, Rubin Institute for Advanced Orthopedics, Sinai Hospital of Baltimore, Baltimore, USA; 2 Baltimore Neurosurgery and Spine Center, Sinai Hospital of Baltimore, Baltimore, USA

**Keywords:** ria, reamer irrigator aspirator, pseudarthrosis, spine fusion, autograft

## Abstract

Background and purpose

Spinal pseudarthrosis (SPA) is a common complication after attempted cervical or lumbosacral spinal fusion surgery. Revision surgeries usually necessitate bone graft implementation as an adjunct to hardware revision. Iliac crest bone graft is the gold standard but availability can be limited and usage often leads to persistent postoperative pain at the donor site. There is scant literature regarding the use of reamer-irrigator-aspirator (RIA)-harvested bone graft in lumbar spinal fusion. This is a collaborative study between orthopedic surgery and neurosurgery departments to utilize femur intramedullary autograft harvested using the RIA system as an adjunct graft in SPA revision surgeries.

Materials and methods

A retrospective review was conducted at a single center between August 2014 and December 2017 of patients aged ≥ 18 years and diagnosed with cervical, thoracic, or lumbar SPA who underwent revision fusion surgery using femur intramedullary autograft harvested using the RIA system. Plain radiographs and CT scans were utilized to confirm successful fusion.

Results

Eleven patients underwent 12 SPA revision surgeries using the RIA system as a source for bone graft in addition to bone morphogenetic protein 2 (BMP-2) and allograft. The mean amount of graft harvested was 51.3 mL (range: 20-70 mL). Nine patients achieved successful fusion (81.8%). The average time to fusion was 9.1 months. Four patients (36.4%) had postoperative knee pain. Regarding patient position and approach for harvesting, 66.7% (n = 8) of cases were positioned prone and a retrograde approach was utilized in 91.7% (n = 11) of cases.

Interpretation

This is the first case series in known literature to report the RIA system as a reliably considerable source of autologous bone graft for SPA revision surgeries. It provides a useful adjunct to the known types of bone grafts. Patient positioning and the approach choice for graft harvesting can be adjusted according to the fusion approach and the surgeon’s preference.

## Introduction

Spinal pseudarthrosis (SPA) is a common complication encountered after attempted cervical or lumbosacral spinal fusion surgery [1]. The increase in spinal fusion procedures performed to treat spinal instability has correspondingly been associated with an increase in the number of revision surgeries needed for SPA [2]. Risk factors for developing SPA include poor bone quality, the existence of multiple medical comorbidities, hardware failure, an imperfect surgical technique including fixation placement, and absence or improper placement of suitable bone graft [3,4]. Although many patients with SPA remain asymptomatic, persistent pain is the most common symptom reported [5]. Clinical assessment and radiographic studies, such as plain radiographs, computed tomography (CT) scans, and magnetic resonance imaging (MRI), are necessary to confirm a diagnosis of SPA [6].

Surgical revision with instrumented fusion is only indicated for symptomatic patients, and the decision to perform the surgery is influenced by many factors, including patient comorbidities, smoking status, SPA location, bone quality, and previously used hardware [1]. Revision usually necessitates bone graft implementation as an adjunct to hardware revision. Types of bone grafts include autografts, allografts, osteoinductive proteins such as bone morphogenetic protein 2 (BMP-2), osteogenic cells such as harvested mesenchymal stem cells, and osteoconductive scaffolds [7]. Among all bone graft options, autograft remains the gold standard due to osteoinductive, osteoconductive, and osteogenic properties, in addition to having no risk for disease transmission. Traditionally, iliac crest bone graft (ICBG) and local laminar or spinous process are the most common autograft donor sites. These options are usually available during primary spine fusions [8]. However, limited availability or insufficiency in case of repeated revisions and persistent postoperative pain at the donor site, in the case of ICBG, can limit their usage [9-12].

The reamer-irrigator-aspirator (RIA) system (DePuy Synthes, West Chester, PA) was originally designed to decrease the pulmonary burden associated with intramedullary (IM) reaming. With its unique feature of combining irrigation and aspiration, the RIA system produces lower IM pressures and lower intra-cortical temperature [13]. Autograft harvest using the RIA technique can offer a greater volume of bone graft with similar union rates and less donor-site pain when compared with autograft obtained from the iliac crest, even if the latter is still available after previous revisions [14-16].

Recent systematic reviews studied the outcomes of various bone grafts compared with bone graft substitutes in spinal fusion procedures, but a review of the literature revealed none that included the use of IM autograft [17,18]. To our knowledge, only one case report described the use of RIA-harvested bone graft in a case of lumbar spinal fusion [19]. The present study represents a collaborative effort between orthopedic surgery and neurosurgery at one institute to utilize the RIA system in SPA revision surgeries.

## Materials and methods

After obtaining an institutional review board exemption, a retrospective review of our institution’s patient records was conducted in accordance with the Helsinki Declaration. All patients aged ≥ 18 years who were diagnosed with SPA (cervical, thoracic, or lumbar) and underwent revision fusion surgeries using femur intramedullary autograft harvested using the RIA from August 2014 to December 2017 were included in this study. Patients whose canal was too small (<12.5 mm) for the RIA were excluded. Patient information, including demographics, body mass index (BMI), Charlson Comorbidity Index (CCI), the number of previously failed SPA revisions, types of other grafts used (if any), patient position during harvest (supine or prone), the approach used (anterograde or retrograde), amount of graft harvested, and the outcome (successful or failed fusion), was identified (Table 1).

**Table 1:** Patient demographic data ACDF, anterior cervical discectomy and fusion; BMI, body mass index; BMP-2, bone morphogenetic protein 2; CCI, Charlson Comorbidity Index; ICBG, iliac crest bone graft; SPA, spinal pseudarthrosis; RIA, reamer-irrigator-aspirator.

Case	Sex	Age (years)	BMI	CCI	Previous constructs that failed	Number of previous revisions for SPA	Levels	Time from the initial procedure (months)	
									1	F	61	31.3	0	L4-L5 decompression and instrumentation		1	21	
									2	F	52	27.5	0	L4-S1 decompression and instrumentation	1	2	19	
3	F	57	31.8	1	L4-S1 decompression and instrumentation		2	8	
4	M	54	22.9	0	C3-C5 ACDF		2	15	
5	F	62	28.2	1	L3-S1 decompression and instrumentation		3	11	
6	F	40	23.6	0	C5-7 ACDF		2	24	
7	M	48	24.5	0	L5-S1 decompression and instrumentation		1	12	
8	F	46	36.5	0	L4-S1 decompression and instrumentation		2	17	
9	M	60	22.7	0	L4-S1 decompression and instrumentation	1	2	32	
					L2-L5 decompression and instrumentation	2	3	24	
10	F	62	29	4	L3-S1 decompression and instrumentation	1	3	20	
11	M	49	21.9	2	L4-S1 decompression and instrumentation	1	2	36	

Diagnosis of SPA was based on the clinical presentation and the imaging studies, after exclusion of other post-fusion causes of persistent pain, and was established by a neurosurgeon after obtaining a detailed history, performing a clinical examination, and obtaining CT scans [20]. Surgical exposure during revision procedures confirmed SPA in all patients. Digitalized plain radiographs were obtained immediately postoperatively and during follow-up visits at two weeks, three months, and six months, while radiographs and CT scans were obtained after six months to confirm fusion. A successful fusion was confirmed using plain radiographs and CT scans by a senior radiologist and the treating neurosurgeon.

After receiving SPA diagnoses and scheduling surgery, patients were referred to the senior author to discuss bone graft using the RIA, including benefits and possible complications [21]. Vitamin D levels were routinely tested in the hospital within the first 12 weeks for all patients. Follow-ups occurred if vitamin D was low or within six months postoperatively. In cases of vitamin D deficiency, a course of 50,000 IU was prescribed once weekly for eight weeks or until the 25-hydroxycholecalciferol level was above 30 ng/ml. Plain radiographs of both femoral bones with magnification markers were obtained to size the IM canal, plan the surgery, and observe any abnormal morphology or hidden pathology.

The senior author does not have a preference regarding patient position or approach when harvesting bone grafts with the RIA system [22]. This decision may be influenced by the preferred starting position of the operating neurosurgeon. The RIA system’s operative technique to harvest autografts from the femoral IM canal has been detailed in the literature [16].

**Figure 1:**
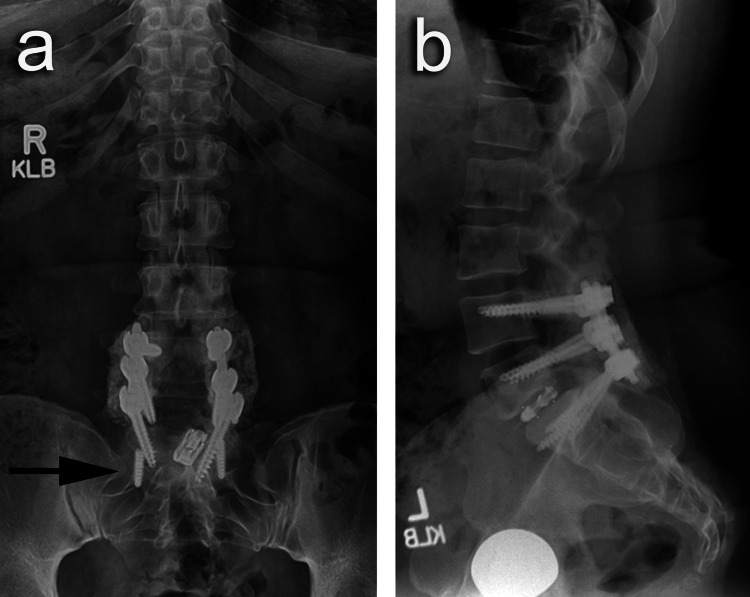
Broken hardware (A) Anteroposterior and (B) lateral preoperative radiographs showing broken hardware with failed fusion.

After successful harvest and subsequent closure of the donor-site incisions, the neurosurgeon proceeded with the revision decompression, instrumentation, and spinal fusion portion of the procedure. All patients received BMP-2 and allograft in addition to the autograft harvested with the RIA system. One case of a smoker who did not achieve fusion after using the RIA graft (Figure 1) was revised with an RIA autograft from the same femur (20 mL) and ICBG. Postoperative radiographs were obtained that demonstrated stable placement of revision instrumentation and bone graft.

## Results

Between 2014 and 2017, 11 patients who underwent 12 revisions using the RIA system were identified, with 63.6% females (n = 7) and 36.4% males (n = 3). Mean ± standard deviation (SD) of age was 53.7 years (range: 40-62 years), BMI was 27.3 ± 4.4 kg/m^2^ (range: 21.9-36.5 kg/m^2^), and CCI average was 0.7 ± 1.2 (range: 0-4). Regarding the levels of presentation, eight cases (66.7%) were lumbosacral, two (16.7%) were lumbar, and two (16.7%) were cervical. The mean number of prior revision surgeries for SPA was 1.3 ± 0.4 (range: 1-2). The time of presentation from the last procedure was 19.9 months (range: 8-36 months).

**Figure 2:**
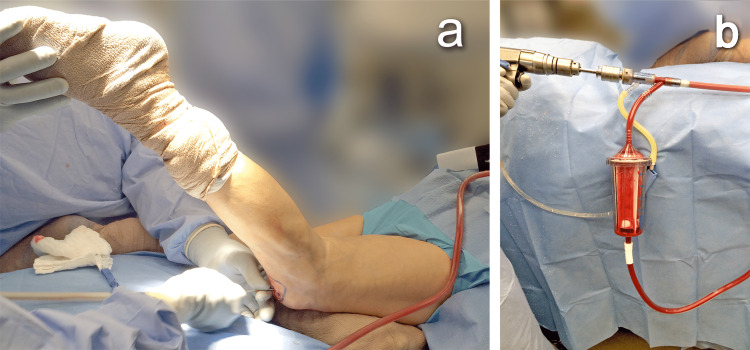
Collecting graft The intraoperative retrograde approach in the prone position (A). The reamer-irrigator-aspirator (RIA) system with the bone graft being collected (B).

Autografts were harvested with the RIA system in the prone position in eight cases (66.7%) and the supine position in four cases (33.3%). Positioning was based upon the neurosurgeon’s preference of approach. The graft was retrieved via a retrograde approach 11 times (91.7%), and the antegrade approach was utilized only in one case (Figure 2). The mean amount of graft harvested was 51.3 ml (range: 20-70 ml). Allograft supplementation and BMP-2 (mean = 3.9 cc; range: 0-8 cc) were used routinely in 90% of procedures. The mean amount of allograft used in revisions was 27.8 cc per case. The amount used was decided intraoperatively, based on the space to be filled.

**Figure 3:**
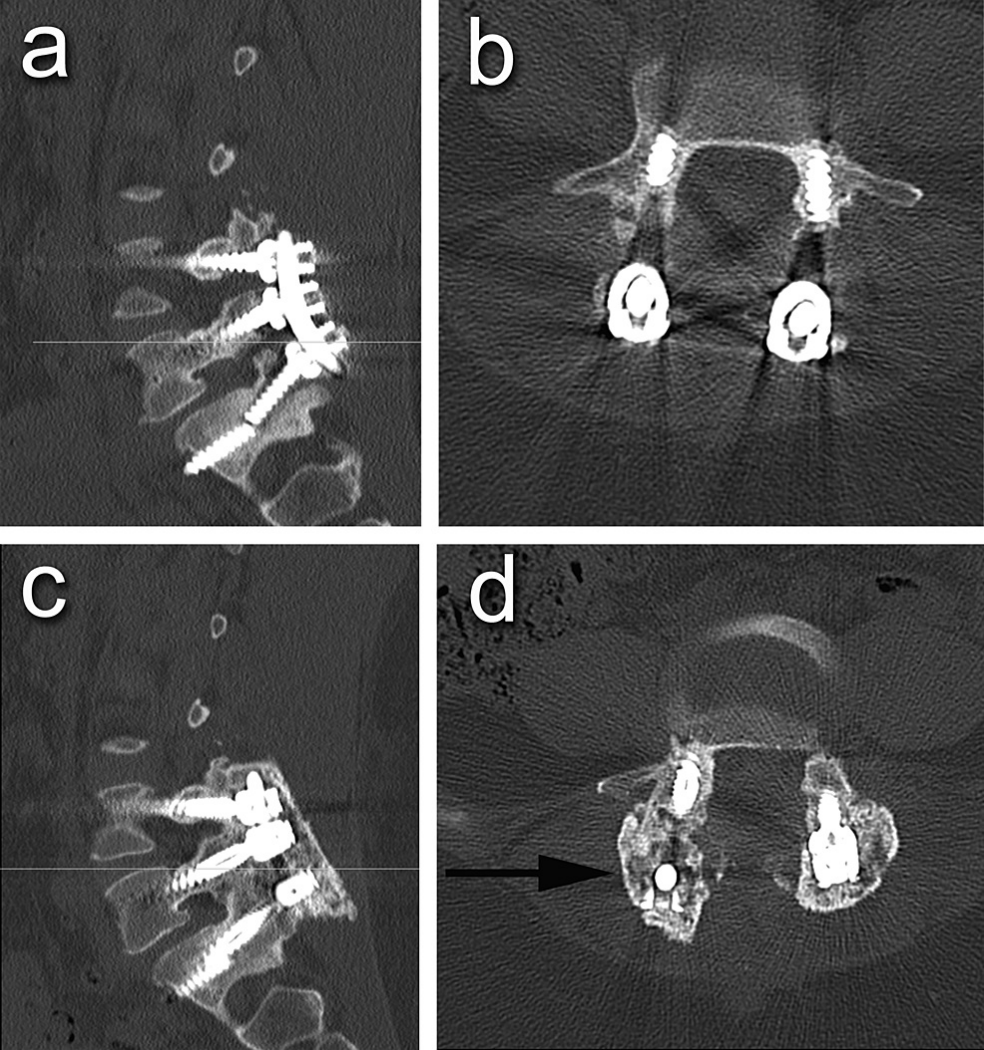
Patient #8 (A) Sagittal and (B) axial views of preoperative CT scan compared with (C) sagittal and (D) axial views of six-month postoperative CT scan.

**Figure 4:**
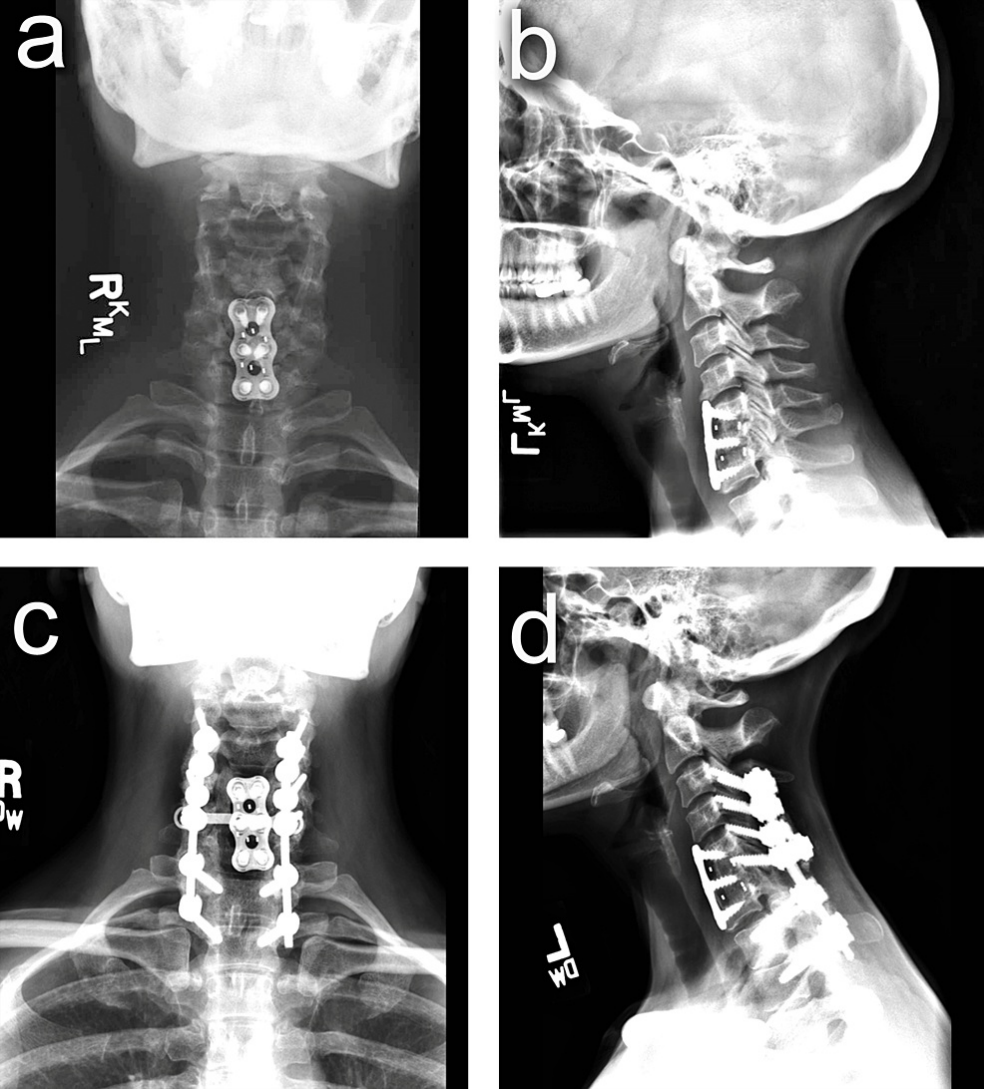
Patient #6 (A) Anteroposterior and (B) lateral preoperative cervical X-rays. (C) Anteroposterior and (D) lateral postoperative cervical X-rays.

**Figure 5:**
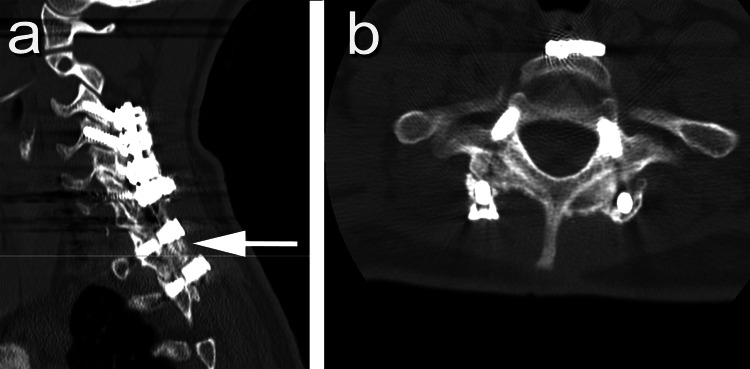
Patient #6 Sagittal (A) and axial (B) cervical CT scans showing successful fusion.

Successful fusion was achieved in 81.8% of patients (n = 9) and in 75% of the revision procedures (Figures 3-5). One patient was infected and did not achieve successful fusion initially; he underwent several debridements to eradicate infection and ended with hardware removal. After infection eradication was confirmed clinically, radiologically, and with negative intraoperative cultures, the revision surgery was performed. Using the RIA system, autograft was harvested from the ipsilateral femur due to inaccessibility of the contralateral femur medullary canal because of hardware and resulted in only 20 mL, so ICBG was performed to achieve the adequate amount of required autograft. In the fused cases, the average time for fusion was 9.1 months (range: 7-12 months) (Table 2).

**Table 2:** Results * The patient had a complex meniscal tear on postoperative MRI. The pain resolved after an arthroscopic partial meniscectomy and chondroplasty of the femoral condyles. ** Infection complicated with vertebral osteomyelitis; the patient underwent multiple revisions to eradicate infection. *** Once the infection was eradicated (confirmed clinically, laboratory, radiologically, and obtained negative intraoperative cultures in the last revision), the patient underwent a successful fusion revision. **** Complicated by ipsilateral knee tense effusion and underwent surgical evacuation of the hematoma on the second day after the fusion. L, left; R, right; RIA, reamer-irrigator-aspirator.

Case	Hardware revision	RIA graft harvesting			Autograft harvested (mL)	Fused	Time to fusion (months)	Follow-up (months)	Knee pain
		Side	Position	Approach					
1	L3-S1 decompression and instrumentation	R femur	Prone	Retrograde	60	No		32	No
2	L4-S1 decompression and instrumentation	L femur	Prone	Retrograde	70	Yes	7	16	Yes*
3	L4-S1 decompression and instrumentation	R femur	Prone	Retrograde	70	Yes	12	5	Yes
4	C3-C7 posterior decompression and instrumentation	R femur	Supine	Antegrade	50	Yes	12	48	No
5	L3-S1 decompression and instrumentation	R femur	Prone	Retrograde	55	Yes	9	21	No
6	C3-T2 posterior decompression and instrumentation	L femur	Supine	Retrograde	45	Yes	9	41	No
7	L4-S1 decompression and instrumentation	L femur	Prone	Retrograde	40	Yes	7	9	No
8	L4-S1 decompression and instrumentation	R femur	Supine	Retrograde	50	Yes	12	39	Yes
9	L2-L5 decompression and instrumentation**	R femur	Supine	Retrograde	50	No		42	No
	T12-L3 decompression and instrumentation***	R femur	Prone	Retrograde	20	Yes	8		
10	Revision and extension of the fusion (T12-S1)	R femur	Prone	Retrograde	55	No		34	No
11	L4-S1 decompression and instrumentation	R femur	Prone	Retrograde	50	Yes	6	14	Yes****

Four patients (36.4%) had postoperative knee pain. The retrograde approach had been utilized in all four surgeries, and three of them were in the prone position (75%). A grade III complex tear in the lateral meniscus was found on a postoperative knee MRI of one patient due to persistent pain. This was managed by arthroscopic partial meniscectomy and chondroplasty of the femoral condyles, and the patient’s knee pain improved during follow-up. This was believed to be an incidental finding and not secondary to the RIA procedure. The last patient with postoperative pain had a tense knee effusion two days after the graft harvesting, which was managed by surgical evacuation of the hematoma.

## Discussion

SPA is a common complication of spinal fusion procedures, affecting up to 23.6% of patients [23]. Several articles studied the outcomes of various bone grafts and bone-graft substitutes in spinal fusion operations in an effort to determine the best method with the least complications [17,18]. Reviewers reported that the ICBG remains the “gold standard” method, owing to its osteogenic, osteoinductive, and osteoconductive properties [17]. Local bone graft is considered to have the same properties as ICBG; however, the limited volume available renders it only an adjuvant to ICBG, especially in multilevel fusion [24,25]. No reviews included bone-graft extraction using the RIA system, potentially because of the lack of reports on its use in this area, even though its use showed promising results for other indications [26-28]. To our knowledge, only a single case report was published describing the RIA system as a bone graft alternative in spinal fusion cases [19].

In our case series, the femur autograft harvested by the RIA was a reliably rich source of autologous bone graft with an average harvest of 51.3 ml. In their cadaveric study, Burk et al. indicated that the average corticocancellous graft provided from the anterior iliac crest was 26.29 mL, the posterior iliac crest yielded 33.82 mL, and the proximal tibia donated 18.11 mL [29].

Despite widespread consensus on the considerable value of ICBG harvesting in the treatment of SPA, donor-site morbidity remains an issue. Complications such as deep infections, vascular injuries, hematoma, and abdominal content herniation from the donor site may occur, necessitating further intervention [10,30,31]. Furthermore, in revision procedures, ICBG is not considered a reproducible source. In contrast, IM bone graft harvested via the RIA system proved to be reproducible with good volume [32], to have growth factors comparable to ICBG [33], and to be an effective harvester of mesenchymal stem cells [21]. Moreover, the RIA system provides the ability to harvest a larger bone graft with less donor-site morbidity and in a shorter operative time [14,15].

In a multicenter randomized controlled trial, Dawson et al. showed that the autograft harvested via the RIA technique had union rates similar to those harvested from the iliac crest with less donor site pain in the treatment of 133 patients with nonunion or traumatic bone defects [15]. RIA was favored in the event that a larger volume of graft would be needed in a shorter harvest time. This advantage can help to avoid the unnecessary increase in the operative duration for the performance of the fusion procedure, which is associated with a wide variety of complications [34].

This study has limitations, namely, the retrospective nature and small sample size presented with the lack of a comparative group. The use of other types of grafts in the revisions (allograft and BMP-2 in all cases and ICBG in one case) may have affected the outcomes, but SPA after spine fusion is a well-known complication that can be devastating and costly, thus combining graft materials and biologics can be essential to achieve successful fusion [35].

The ability to harvest a reproducible source of bone graft while the patient is in the prone position is considered a great advantage of the RIA technique. However, it was noted that among the four patients who had postoperative knee pain, three were positioned prone. We theorize that the continuous pressure placed on the knee during the lengthy spine procedures may be the cause of the postoperative pain, but that position was decided according to the neurosurgeon's preferred approach. However, the small sample size of this study does not allow confirmation of this theory. Recently, Brewster et al. showed no difference in the long-term patients’ functional outcomes of femur nailing using the antegrade or retrograde approach, which can be decided according to the surgeon's preference for the best accessibility to the femur IM canal in each patient [22]. Lastly, the RIA technique is not free of complications, including postoperative knee pain. Other studies reported “excessive” postoperative pain, cortical perforations, fractures, and pulmonary embolism [36-39].

## Conclusions

The RIA system has been described in the literature as a reliable source of autograft that shares the advantages of ICBG, but to our knowledge, it has not previously been reported in the treatment of SPA. This is the first case series to report the femur intramedullary autograft harvested using the RIA as a reliable, reproducible, and rich source of an adjunct autologous bone graft in the revision of SPA. Postoperative knee pain is a possible complication. The approach and patient’s position utilized can be varied in accordance with the patient’s condition, surgery requirements, and surgeon’s preference.
